# *In-vivo* studies of targeted and localized cancer drug release from microporous poly-di-methyl-siloxane (PDMS) devices for the treatment of triple negative breast cancer

**DOI:** 10.1038/s41598-023-50656-6

**Published:** 2024-01-02

**Authors:** S. C. Eluu, J. D. Obayemi, A. A. Salifu, D. Yiporo, A. O. Oko, T. Aina, J. C. Oparah, C. C. Ezeala, P. O. Etinosa, C. M. Ugwu, C. O. Esimone, W. O. Soboyejo

**Affiliations:** 1 Department of Pharmaceutical Microbiology and Biotechnology, Nnamdi Azikiwe University, Ifite Awka, 420110 Anambra State Nigeria; 2 Department of Mechanical Engineering, Higgins Lab, Worcester Polytechnic Institute (WPI), 100 Institute Road, Worcester, MA 01609 USA; 3 Department of Biomedical Engineering, Gateway Park Life Sciences and Bioengineering Centre, Worcester Polytechnic Institute, 60 Prescott Street, Worcester, MA 01609 USA; 4 Department of Engineering, Morrissey College of Arts and Science, Boston College, Boston, USA; 5 Department of Mechanical Engineering, Ashesi University, Berekuso, Ghana; 6 Department of Biology and Biotechnology, David Umahi Federal, University of Health Sciences, Uburu, Nigeria; 7 Department of Material Science, African University of Science and Technology, Km 10 Airport Road, Abuja, Nigeria; 8 Department of Engineering, SUNY Polytechnic Institute, 100 Seymour Rd, Utica, NY 13502 USA

**Keywords:** Breast cancer, Drug delivery, Biological techniques, Biophysics, Drug discovery, Oncology

## Abstract

Triple-negative breast cancer (TNBC) treatment is challenging and frequently characterized by an aggressive phenotype and low prognosis in comparison to other subtypes. This paper presents fabricated implantable drug-loaded microporous poly-di-methyl-siloxane (PDMS) devices for the delivery of targeted therapeutic agents [Luteinizing Hormone-Releasing Hormone conjugated paclitaxel (PTX-LHRH) and Luteinizing Hormone-Releasing Hormone conjugated prodigiosin (PG-LHRH)] for the treatment and possible prevention of triple-negative cancer recurrence. In vitro assessment using the Alamar blue assay demonstrated a significant reduction (p < 0.05) in percentage of cell growth in a time-dependent manner in the groups treated with PG, PG-LHRH, PTX, and PTX-LHRH. Subcutaneous triple-negative xenograft breast tumors were then induced in athymic female nude mice that were four weeks old. Two weeks later, the tumors were surgically but partially removed, and the device implanted. Mice were observed for tumor regrowth and organ toxicity. The animal study revealed that there was no tumor regrowth, six weeks post-treatment, when the LHRH targeted drugs (LHRH-PTX and LHRH-PGS) were used for the treatment. The possible cytotoxic effects of the released drugs on the liver, kidney, and lung are assessed using quantitative biochemical assay from blood samples of the treatment groups. Ex vivo histopathological results from organ tissues showed that the targeted cancer drugs released from the implantable drug-loaded device did not induce any adverse effect on the liver, kidneys, or lungs, based on the results of qualitative toxicity studies. The implications of the results are discussed for the targeted and localized treatment of triple negative breast cancer.

## Introduction

The most common cancer among women and the leading cause of cancer death worldwide is breast cancer^1^. Breast cancer is divided into five typical molecular characteristics based on the expression of progesterone receptor (PR), estrogen receptor (ER), and human epidermal growth factor receptor 2 (HER2)^2^. These categories are luminal A (ER + , PR + , and HER2-), luminal B (ER + /PR + , and HER2 +), HER2 positive (ER/PR, and HER2 +), triple-negative/basal-like (ER-, PR-, and HER2-), and claudin-low^3^.

Triple-negative breast cancer (TNBC) is defined by the absence of three breast cancer markers: ER, PR, and HER2 expression. Triple-negative (TN) tumors are often larger and have a higher grade at presentation than other breast cancers. They are also associated with aggressive clinical behavior, which frequently results in early metastasis, particularly to visceral locations^4^. As a result, TNBC is linked with a poor prognosis^5^. Due to the disease's heterogeneity and the lack of well-defined molecular markers, the treatment of this subtype of breast cancer has been very problematic^6^. Although traditional treatment strategies are effective to some extent, significant limitations such as medication accessibility to distant tumor cells, greater therapeutic doses, nonspecific targeting, uneven biodistribution, and undesired side effects limit the efficiency of current triple negative breast cancer therapy^7^.

Targeted chemotherapy is a new technique for systemic chemotherapy^8^. It aims to improve the efficacy of drug delivery while reducing the side effects^9^. Targeted drug delivery is the transfer of drugs in a manner that increases the concentration in some parts of the body relative to others. A ligand for certain receptors that are expressed selectively on tumor cells is attached to a drug to achieve targeting^10^.

One of the methods that is used for the engineering of targeted cancer therapy is based on the discovery that tumors produce higher levels of receptors for peptide hormones such as somatostatin, bombesin, and LHRH (now identified in genome and microarray databases as GNRH1) than most normal cells^11,12^. The past decade has witnessed the explosive development of targeted drug delivery systems for the diagnosis and treatment of cancer^1,13,14^, among which Luteinizing Hormone Releasing Hormone-conjugated drugs (LHRH-paclitaxel and LHRH-prodigiosin) have attracted much interest^15–18^. These conjugated drugs hold great promise for the treatment of TNBC. Prior results from these studies have shown that the conjugation of LHRH to paclitaxel or prodigiosin increases the effectiveness of these drugs in the treatment of TNBC. The specific targeting of the LHRH receptors by the LHRH peptide enhances the specific targeting of breast tumor cells/tissues. This ultimately results in the shrinkage and elimination of triple negative breast tumor xenografts. It also provides benefits such as improved therapeutic effects and enhanced targeting via the enhanced permeability effect.

The LHRH receptor (LHRH-R) belongs to the superfamily of G protein-coupled receptors^19^, which is overexpressed in several types of human cancer cells, including ovarian, breast, and prostate cancer cells^20,21^. LHRH is a good candidate for conjugation because of its non-toxic and non-immunogenic properties. It also has a flexible, highly water-soluble chain that can extend to produce native lysine ε-amine groups that can be utilized for drug coupling. Hence, the conjugation of LHRH to cancer drugs is an effective strategy for increasing the efficacy of cancer drugs while reducing the toxicity associated with cancer treatment. However, although prior studies have provided insights into the efficacy of LHRH-conjugated cancer drugs, there have been only limited studies of the toxicity associated with such localized cancer treatments^22^. Since most drugs fail during phase III clinical trials due to efficacy issues and toxicity^23^, there is a crucial need to study the possible toxicity that is associated with LHRH-conjugated cancer drugs such as LHRH-paclitaxel and LHRH-prodigiosin. There is also an opportunity to enhance the efficacy of such drugs by delivering them locally from drug delivery systems^24^.

Thus, the objective of this in vitro and in vivo study is to explore the efficacy of drug-loaded microporous PDMS implantable devices for the targeted and localized delivery of LHRH-conjugated cancer drugs (paclitaxel and prodigiosin). Additionally, the potential to enhance the therapeutic effectiveness of unconjugated prodigiosin and paclitaxel is explored through the utilization of this microporous PDMS device. The ability to prevent tumor re-occurrence after the excision of TNBC xenograft tumors was explored and the kinetics of drug release were studied as well. The effects of the cancer drugs on the liver, kidney, and lungs of nude mice that were used in in vivo models of the study were assessed to understand the safety profile of targeted and untargeted drug delivery. The implications of the results are discussed for the development of novel devices for the targeted and localized treatment of triple-negative breast cancer.

## Results and discussion

### FTIR analysis on PDMS-based substrates

**Figure 1:**
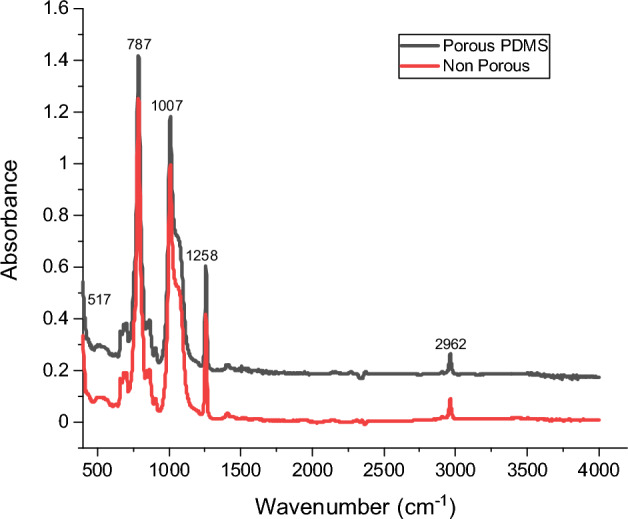
FTIR spectra of porous and non-porous PDMS.

FTIR was used to identify and characterize unknown materials, find additives, detect impurities in a material, and determine decomposition and oxidation^25^. Figure 1 presents the FTIR spectrum of porous and non-porous PDMS. FTIR analysis was used to verify the molecular structures of porous and non-porous PDMS. When FTIR spectra for porous and non-porous PDMS were compared to see whether porosity affected the properties of PDMS, the results showed that there was no observable difference between the two. The typical IR bands could be seen in the infrared (IR) spectra of the different PDMS polymers (Fig. 1).

The distinctive bands of the polymer were seen using FTIR spectroscopy at wavenumbers of 2962 cm^−1^ (stretching of C–H in CH_3_), 1258 cm^−1^ (symmetric CH_3_ bending in Si-CH_3_), 1076 cm^−1^, and 1007 cm^−1^ (Si – O—Si), and at 787 cm^−1^ (rocking of CH_3_ in Si-CH_3_). The characteristic bands observed in this study are consistent with the findings from past investigations^26–29^. This result shows that there was no difference between the characteristic pattern of absorption bands of the porous and non-porous PDMS, implying that the creation of pores did not alter the material composition in any way. It was also noticed that there were no new peaks observed, suggesting that the sugar that was used to create the pores was eliminated during leaching.

### Morphological analysis of PDMS surfaces

**Figure 2:**
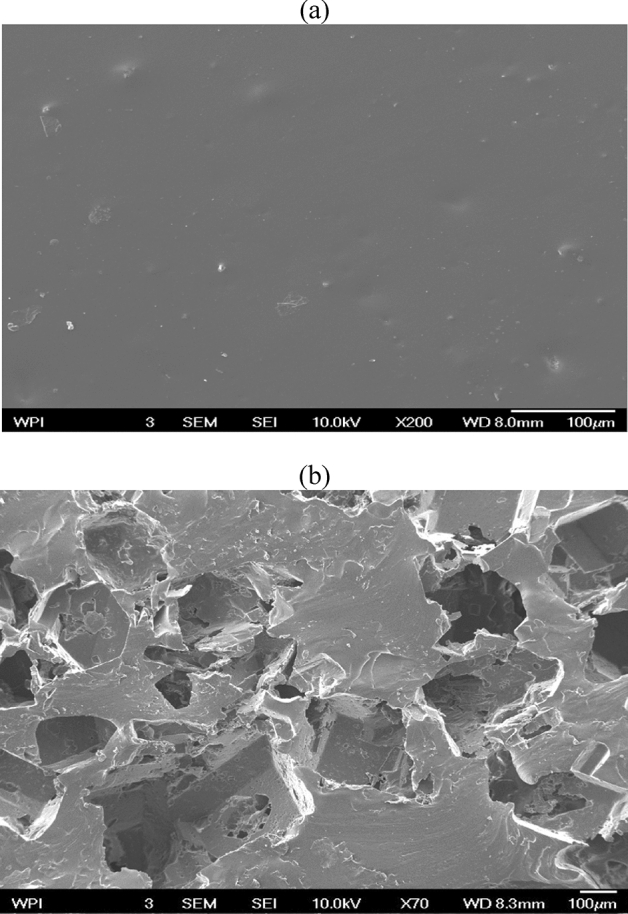
Morphological images of PDMS surfaces at X 70 and X 200 magnification: (**a**) nonporous PDMS; and (**b**) porous PDMS made from sugar cubes.

Scanning electron microscopy (SEM) was used to characterize the morphologies of the non-porous and porous PDMS (which was fabricated via solvent casting using the sugar templating process). The resulting images are presented in Fig. 2. The non-porous and porous PDMS are shown in Fig. 2a,b, respectively. The differences in morphology between porous and non-porous PDMS are visible in the SEM micrographs of the PDMS polymer. The observed morphology in the porous PDMS indicates that a homogeneous porosity was produced by using granulated sugar as the porosity-creating agent.

In terms of the pore layout, the microstructure created within the network can be defined as highly consistent and regular. The porous PDMS layers successfully mimicked the structure of the sugar cube. On the PDMS surface, pores with an approximately cubic form were randomly dispersed, and the diameter of the pores was similar to that of the sugar cube templates. In addition, it is interesting to note that the non-porous PDMS did not form any pores, unlike the other porous polymers. The non-porous polymer was free of cracks and porosity (Fig. 2b). PDMS of similar porosity has also been fabricated from different powder templates^30,31^.

### Thermo-gravimetric analysis of PDMS-based substrates

Thermo-gravimetric analysis (TGA) was used to investigate the thermal stability and decomposition of polymeric materials^32^. The amount of mass lost because of thermal events, such as the degradation of polymer components, is presented on a plot of mass as a function of temperature. TGA was used to determine the thermal characteristics of the PDMS samples heated from about 25 to 800 °C at a heating rate of 20 K/min under a nitrogen gas flow rate of 20 mL/min. This was used to maintain a non-reactive atmosphere as the analysis progressed.

**Figure 3:**
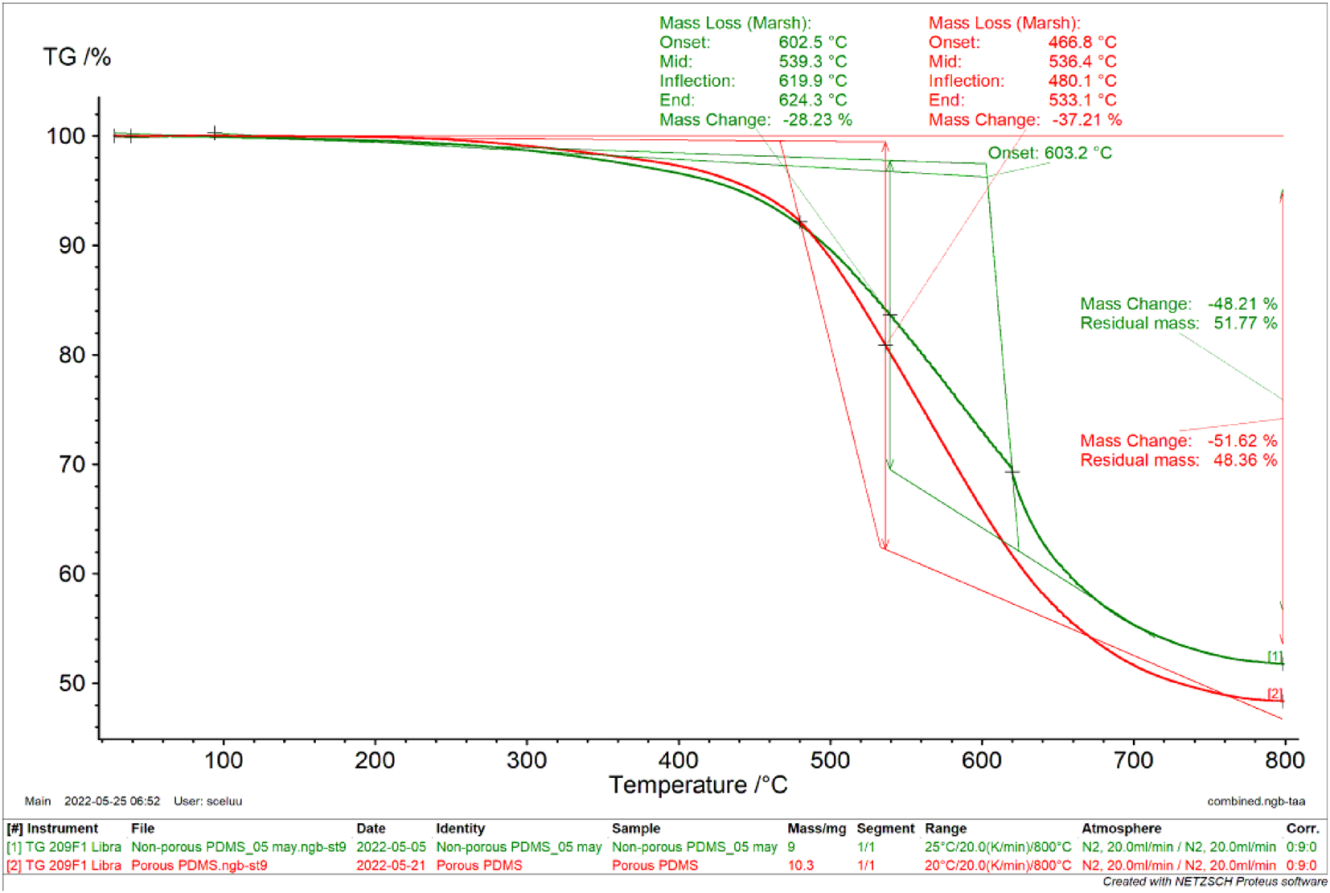
Thermogravimetric analysis (TGA) curves of porous and non- polydimethylsiloxane (PDMS).

The thermal stability curves of the porous and non-porous PDMS analyzed (Fig. 3) were almost identical, regardless of the porosity of the porous PDMS. This is because both porous and non-porous PDMS were formed of the same components, with the exception that sugar was used to generate pores in the porous PDMS, which was entirely leached out of the substance during the soaking times. The PDMS curve shown in this work is comparable to that described in other investigations^33–36^. Both porous and non-porous PDMS degraded thermally more slowly over a larger temperature range, with the initial breakdown temperature rising as the temperature rose. The porous and non-porous PDMS underwent 51.62 and 48.21% total mass loss up to 800 °C, respectively. The lower mass loss of the porous device is attributed to the porous structure, which limits the thermal conductivity and the mass loss.

### Differential scanning calorimetry analysis

The DSC was used to measure the physical and chemical changes within the PDMS device in response to temperature. Some of the specific information obtained from DSC measurements includes glass transition temperatures, melting exothermic transitions, recrystallization, specific heat capacity, etc. The porous materials had a specific heat capacity of 0.117 J/gK, compared to 0.089 J/gK for the non-porous material. The higher specific heat capacity of the porous material suggests that more heat was required to cause an increase in temperature. This is due to the porous material's hollow structure, which, in contrast to the non-porous material, prevented it from effectively retaining heat and caused it to react poorly to changes in temperature and the application of heat. Each state of matter has a unique specific heat capacity that changes with temperature. The DSC data further reveals the glass transition temperature in a certain temperature region in which the heat curve deviates from linear behavior.

**Figure 4:**
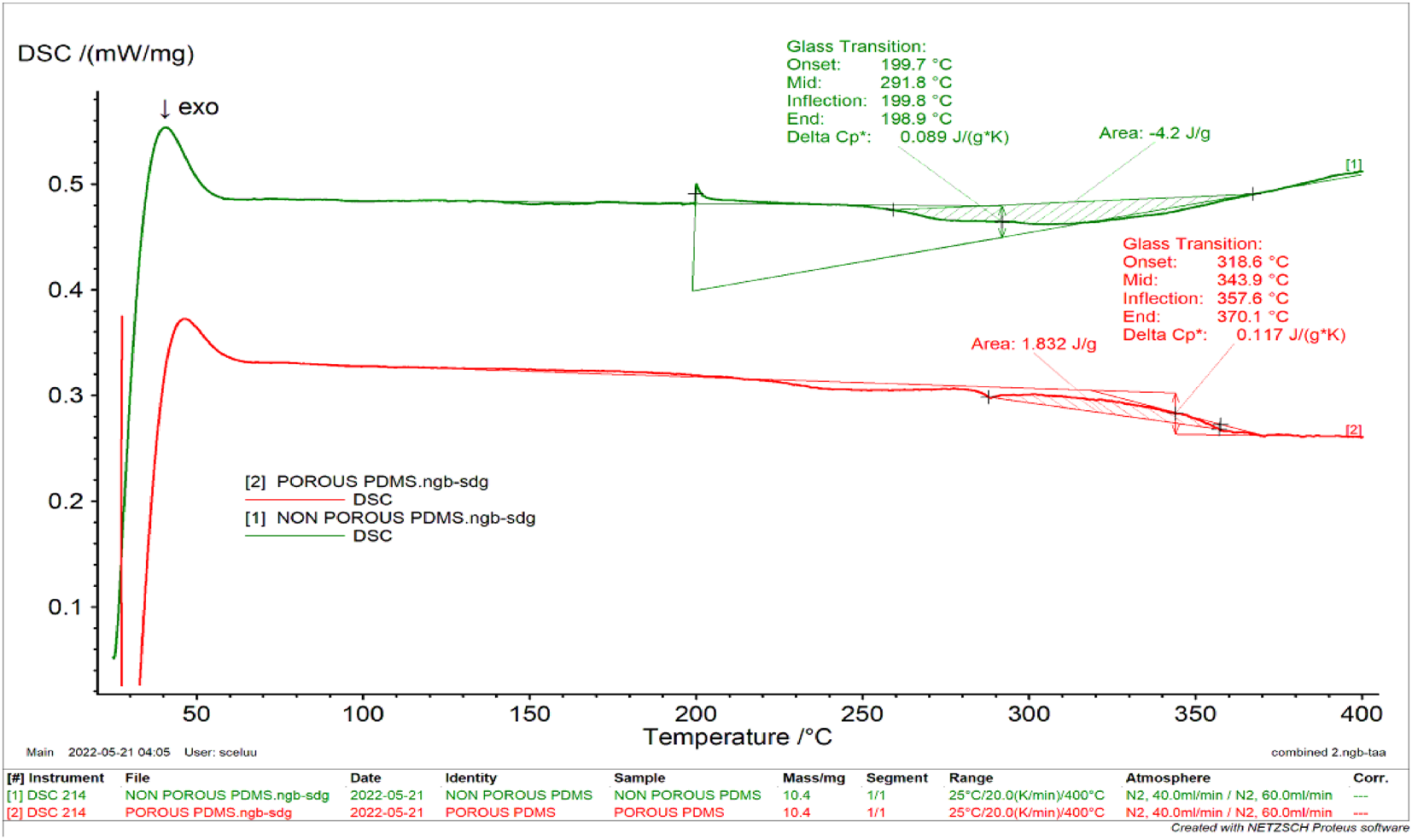
Differential scanning calorimetry (DSC) thermograms of porous and non-polydimethylsiloxane (PDMS).

For the porous PDMS, the onset, mild, inflection, and end temperatures were 199.7, 291.8, 199.8, and 198.9 °C, respectively (Fig. 4). However, the onset and end temperatures of the non-porous PDMS were found at T_go_ = 318.6 °C and T_ge_ = 370.1 °C, respectively. The inflection temperature in between was 357.6 °C while the mid-temperature was 343.9 °C. The DSC result further showed a shift of the baseline around 50 °C for both the porous and non-porous PDMS, indicating a glass transition. Moreover, a peak of exothermic activity was seen for the porous PDMS at a temperature of around 310 °C, pointing to an exothermic process brought on by crystallization. With the non-porous PDMS, an endothermic reaction by melting is indicated by an endothermic peak that was seen around 330 °C. The outcome demonstrates the substantial temperature dependence of porous PDMS crystallization. Slow cooling promotes the production of stable DSC crystals with higher melting points and fusion enthalpies because it causes crystallization to occur at higher temperatures^37^. The overall area under the curve of the porous PDMS was somewhat higher than that found for the non-porous PDMS sample.

### In vitro drug release

**Figure 5:**
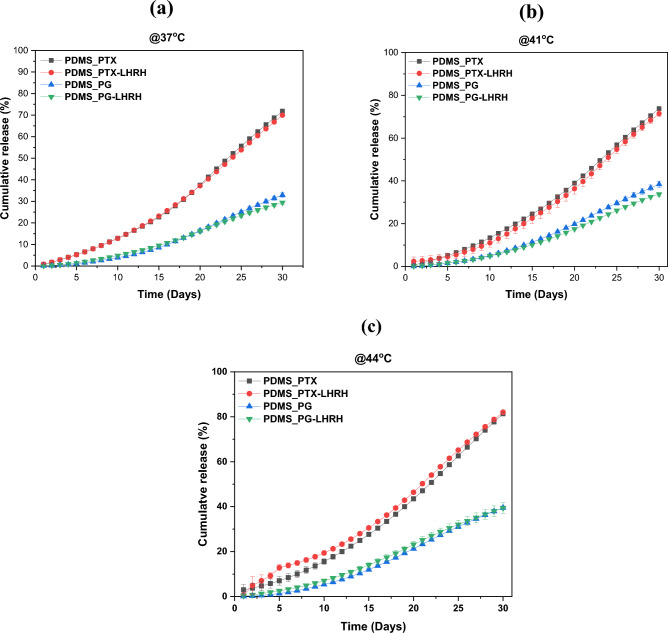
Cumulative drug release from microporous PDMS device for 30 days in a buffer of pH 7.4 at different temperatures (**a**) 37 °C, (**b**) 41 °C, and (**c**) 44 °C. Each line on the graph corresponds to different formulations, including PDMS_PTX, PDMS_PTX-LHRH, PDMS_PG, and PDMS_PG-LHRH.

Temperature-responsive or sensitive polymers can be used to modulate drug release rates in various drug delivery systems. Temperature-controlled drug release enables targeted administration of drugs to certain tissues or areas. This can reduce systemic side effects and increase the therapeutic efficacy of drugs. The results of in vitro release of PTX, PTX-LHRH, PG, and PG-LHRH from a PDMS microporous device at body temperature (37 °C) and within the hyperthermic range (41 and 44 °C) are presented in Fig. 5. At 37 °C, the device released 72 and 70% of PTX and PTX-LHRH over the course of 30 days, compared to approximately 33 and 29% of PG and PG-LHRH during the same time. At 41 °C, 74 and 72% of the PTX and PTX-LHRH drugs, respectively, were released after 30 days. For PG and PG-LHRH, 38 and 34% of drugs were released at 41 °C, respectively. Then, at 44 °C, approximately 40 and 39% of the PG and PG-LHRH, as well as 81 and 82% of the PTX and PTX-LHRH, were released, respectively.

The slow release of PG and PG-LHRH compared to PTX and PTX-LHRH is because prodigiosin and paclitaxel have different physicochemical properties^38^. This may be connected to the molecular weight of prodigiosin^39^ and that of paclitaxel (853.9 g/mol). Molecular weights have been shown to influence the rate of diffusion of drugs through the drug delivery system, leading to slower drug release^38^. The results obtained indicate that there was a prolonged and sustained release of the drugs over a period of thirty days. A regular pharmacological dosage within the therapeutic window is essential to achieve the desired therapeutic index^40^. Conventional drug delivery has several major limitations, including inadequate bioavailability, large dose requirements, unfavorable side effects, low therapeutic indices, the development of multiple drug resistance, and non-specific targeting^41^. However, due to the observed prolonged release, this drug delivery system could potentially be favorable for clinical anticancer therapy by lowering the frequency of administration.

Furthermore, a substantially higher cumulative release was generally seen at higher temperatures of 41 and 44 °C, which are within the hyperthermic range (41–44 °C), with the highest cumulative release observed at 44 °C. Similar studies have also reported an increase in drug release with an increase in temperature^42–46^. Quick onset of action is one of the clinical benefits of a higher drug release rate at higher temperatures. For example, a higher release rate at higher temperatures could lead to increased drug availability at the tumor site. This could lead to improved therapeutic efficacy, especially in cases where obtaining a rapid and significant drug concentration is essential for the best possible treatment outcome. When patients experience faster relief or see more immediate results from their medication, it can improve their adherence to the treatment plan. This is especially relevant for chronic conditions, where patients may be more likely to stick to their medication regimen if they notice quick improvements.

### *In-vitro* drug release rate

The correlation coefficients R^2^ that were determined for the release kinetics are shown in Table 1. The release kinetics for the PDMS drug delivery system that has the highest R^2^ correlation coefficient value satisfy the Korsmeyer-Peppas model^47^. The PDMS drug delivery system's release exponent range is consistent with anomalous transport at the three different temperatures taken into consideration in this work. The value of n (Kosmayer-Peppas model) obtained for both the conjugated and non-conjugated drug PDMS delivery systems at 37, 41, and 42 °C (Table 1) is similar to the model drug release mechanism reported by Ahmed et al^48^, indicating non-Fickian (anomalous) release behavior. This mechanism of release could be attributed to a combination of both diffusion and erosion-controlled drug release, which could be mediated by the rapid hydration, swelling, and erosion of polymeric blends^47,49^.

**Table 1:** Korsmeyer and Peppas model drug release mechanism.

	Intercept (lnk)	Slope (n)	R^2^	Drug release model	Temperature
PDMS_PTX	− 0.4354	0.57147	0.90762	Anomalous (non-Fickian Diffusion)	37 °C
PDMS_PTX-LHRH	− 0.47175	0.55651	0.95423	Anomalous (non-Fickian Diffusion)	
PDMS_PG	− 0.09488	0.9946	0.9447	Anomalous (non-Fickian Diffusion)	
PDMS_PG-LHRH	− 0.0048	0.76398	0.88686	Anomalous (non-Fickian Diffusion)	
PDMS_PTX	− 0.20345	0.56971	0.9615	Anomalous (non-Fickian Diffusion)	41 °C
PDMS_PTX-LHRH	− 0.58549	0.92449	0.96463	Anomalous (non-Fickian Diffusion)	
PDMS_PG	0.47894	0.85051	0.9538	Anomalous (non-Fickian Diffusion)	
PDMS_PG-LHRH	0.09718	0.77487	0.94498	Anomalous (non-Fickian Diffusion)	
PDMS_PTX	0.51071	0.65759	0.98333	Anomalous (non- Fickian Diffusion)	44 °C
PDMS_PTX-LHRH	− 0.98913	0.84079	0.90182	Anomalous (non- Fickian Diffusion)	
PDMS_PG	0.11451	0.89805	0.91771	Anomalous (non- Fickian Diffusion)	
PDMS_PG-LHRH	− 0.27714	0.56684	0.85906	Anomalous (non-Fickian Diffusion)	

### Cytotoxicity of drugs delivered from the microporous device

Using the Alamar Blue assay, the cytotoxic effects of different treatments on the cultured MBA-MD-231 (cancer) breast cell lines were examined. In group one, different drugs were administered to the cell lines via microporous devices. This was done for durations of 0, 6, 24, 48, 72, and 96 h. The outcomes demonstrated a significant reduction (P < 0.05) in percentage cell growth inhibition in a time-dependent manner in the groups treated with PG, PG-LHRH, PTX, and PTX-LHRH. The reduction in the percentage Alamar Blue in the treated cells may be associated with the therapeutic effects of the drugs, which altered the metabolic activity of the cells, leading to inhibition of proliferation or induction of apoptosis. Moreover, the reduction in cell growth in a time-dependent manner shows that longer treatment durations are associated with increased treatment effectiveness. This implies that the drugs require sufficient exposure time to exert maximum inhibitory effects. However, group two, which corresponds to microporous devices without drugs, exhibited no growth inhibition during the period of the study.

**Figure 6:**
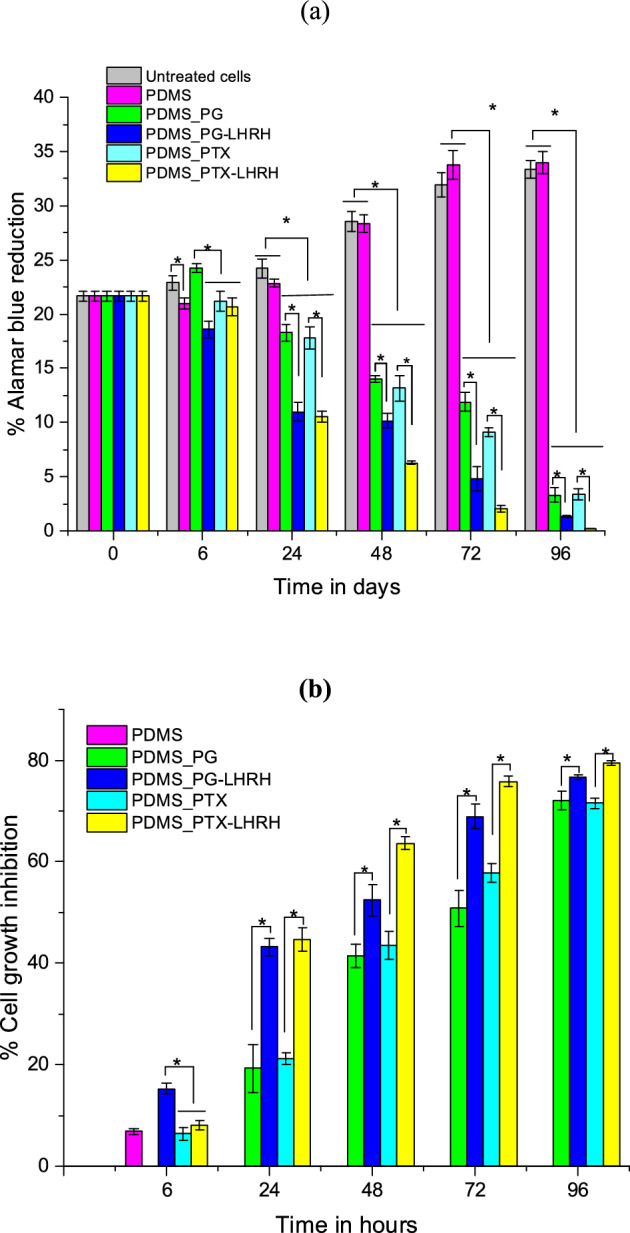
Cytotoxicity of different drugs delivered from the microporous device in MDA-MB-231 cells by Alamar blue assay (**a**) percentage Alamar blue reduction (**b**) percentage cell growth inhibition. Asterisk (*) shows that the mean difference between groups is significant at P < 0.05.

At 48 and 72 h, the group treated with PTX-LHRH showed a significantly higher reduction (P < 0.05) in percentage cell growth inhibition as compared to the group treated with PG-LHRH; nevertheless, at 24 and 96 h, there was no significant difference (P > 0.05) in percentage cell growth inhibition between the two groups. However, during the evaluation periods, the PG-LHRH and PTX-LHRH groups significantly reduced (P < 0.05) the percentage of cell growth inhibition more than any other group, including the PDMS (no drug), PG, and PTX groups. The percentage reduction in Alamar Blue for the treated cells was also determined (Fig. 6a). Similarly, the result of the percentage Alamar Blue reduction, which corresponds to cell viability, showed that the cell survival rate was significantly decreased (P < 0.05) for the groups treated with the conjugated drugs compared with the unconjugated drugs after delivery for 24, 48, 72, and 96 h. In general, the result of the percentage reduction in Alamar Blue (Fig. 6a) followed the same trend as the percentage cell growth inhibition result (Fig. 6b) throughout the periods of the study.

According to the results of the study, MBA-MD-231 cells were found to be more sensitive to the cytotoxic effects of the conjugated drugs. Moreover, in all the experiments, the conjugated drugs achieved better efficacy than each drug alone, whereas the effects of the conjugated drugs on the biochemical and histological parameters remained similar to those when the drug was administered alone. Hence, the current results are well aligned with the growing body of scientific evidence that suggests that LHRH can serve as targeting agents for the specific targeting and localized treatment of breast cancer^50–53^. This may be attributed to smart drug delivery, which is expected to increase the concentration of the drug in the tumor site. Targeted drug delivery techniques concentrate medication in specific body areas. From Paul Ehrlich perspective when he introduced the concept of "magic bullets" over a century ago to selectively target pathogens without harming the host organism, our approach holds promise for minimizing off-target effects and reducing toxicities of drug.

Furthermore, the percentage reduction in Alamar Blue results indicated that the group that had the device, but no drug could not halt the survival of the MBA-MD-231 cells. It was also noted that the untreated cells had a significantly higher Alamar Blue reduction (P < 0.05) throughout the experiment, which suggests increased metabolic activity.

### Effect of treatments on the induction of apoptosis

The mechanism of natural and programmed cell death called apoptosis is distinguished by several morphological characteristics that are common to it. These include cell shrinkage; fragmentation into membrane-bound apoptotic particles, and fast phagocytosis by nearby cells^54^. Numerous substances, such as mild doses of radiation, hypoxia, heat, cytotoxic medicines, or more specialized anti-cancer chemicals, can cause an organism to undergo apoptosis^55^.

The stimulation of apoptotic pathways is one crucial way in which cytotoxic drugs eliminate cancer cells^56^. To determine if the growth-inhibitory activity seen after the treatment of MDA-MB-231 cells as observed in this study was caused by the induction of apoptosis, different treatments, such as PG, PG-LHRH, PTX, and PTX-LHRH were delivered to the cells for 24 h and subsequently stained with Annexin and propidium and analyzed using a flow cytometer.

**Figure 7:**
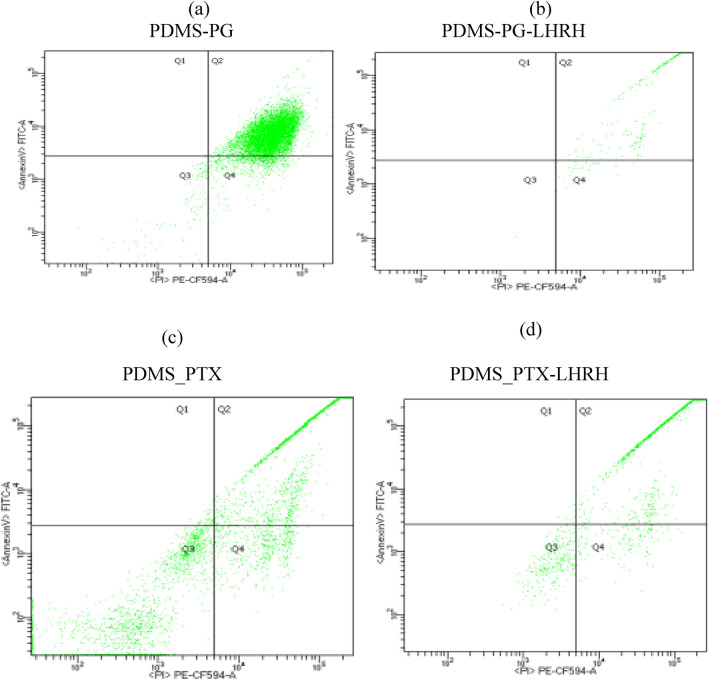
Induction of apoptosis in MDA-MB-231 cells after exposure to different treatments including (**a**) PG, (**b**) PG-LHRH, (**c**) PTX, and (**d**) PTX-LHRH and incubated for 24 h at 37 °C in a CO_2_ incubator. Cells were examined by flow cytometry after being stained with annexin and propidium.

The early apoptotic stage (AV + /Pi −) is displayed in the lower right quadrant of each panel, while the late-stage apoptosis/cell death is shown in the upper right quadrant, as observed in Fig. 7. The result of the study showed that, in all treatment groups (PG, PG-LHRH, PTX, and PTX-LHRH), apoptosis was detected, implying induced cell death in MDA-MB-231 cells. Furthermore, it was observed that both unconjugated and conjugated drugs delivered from the microporous device induced early and late apoptosis in tumor cell lines exposed for 24 h. These findings support the potential therapeutic effects of LHRH-conjugated prodigiosin and paclitaxel drugs on triple negative breast cancer cells.

Prior work has demonstrated that the activation of apoptosis by paclitaxel appears to be the primary mechanism by which cancer cells are killed^57,58^. Furthermore, convincing evidence suggests that paclitaxel induces cytotoxicity by predominantly affecting the cytoskeleton's microtubule components, leading to the arrest of the mitotic stage^58–60^. Paclitaxel stimulates microtubular connections to activate the c-Jun N-terminal kinase/stress-activated protein kinase (JNK/SAPK)1 pathway in a range of human cells^61^.

Different reports confirm our findings, demonstrating that prodigiosin causes apoptosis in various cellular models^62–67^. According to these reports, the control of the mitochondrial apoptosis pathway plays a major role in the PG-induced death of tumor cells^68^. By activating caspase9 to remove caspase3 from the mitochondrial apoptosis pathway, cytochrome C can cause apoptosis^69^. Prodigiosin also causes DNA damage, which then induces the accumulation of p53^70^. Other studies have suggested that chemotherapeutic drugs for cancer cause DNA damage in some cells, which can cause apoptosis through a p53-dependent mechanism^71^. The p53 protein's ability to boost the expression of the p21 gene is its primary biological impact. By keeping cells in the G1 phase from moving into the S phase during the cell cycle, the p53 protein repairs damaged DNA or chromosomes by inducing apoptosis, which helps to remove damaged cells to regulate and promote the function of chemotherapy agents^65,72^.

The p53 protein interacts with two distinct protein classes: the pro-apoptotic (Caspases Family; Bcl-2; Bid that encourages the release of cytochromes C from the mitochondria to the cytoplasm) and the anti-apoptotic (IAPs) that inhibit the caspases and so prevent the induction of apoptosis ^64^. In a different mechanism, the cell is killed by perforin-granzyme-dependent cytotoxicity and T-cell-mediated cytotoxicity. Granzyme B or granzyme A are two ways that the perforin/granzyme pathway might cause apoptosis^71^. In addition, numerous routes that might result in apoptosis have been suggested. Caspase-3 and the Bcl-2 family are crucial apoptosis regulators^73^. Upregulation of Bax and downregulation of BcI-XL are linked to the Bcl-2 proteins^74^.

### In vivo studies

Compared to other kinds of breast cancer, triple-negative breast cancer can spread more quickly and is more inclined to recur^75^. Even after breast-conserving surgery, it is still possible for cancer cells to remain in the affected area. Such residual tumor cells can proliferate and ultimately result in tumor recurrence and/or metastasis^76^. Hence, chemotherapy following surgery is common and essential. Systemic chemotherapy, however, has several shortcomings. These include high dosages, hazardous side effects, and frequent treatments^77^. The use of implantable biomedical devices offers several advantages, including sustained and controlled drug release, minimization of drug level fluctuations, extended duration of action, and reduced side effects^77,78^. Hence, in this study, we delivered PG, PTX, PG-LHRH, and PTX-LHRH to the tumor site after surgery via implantable microporous PDMS devices.

Our results show that the porous PDMS drug delivery system effectively inhibits triple negative breast cancer recurrence and metastasis assessment. Thus, no tumor regrowth was observed (after 6 weeks of drug elution) in the groups that had the drug-eluting devices. In contrast, there was tumor regrowth in the control group, which had neither the implant nor anything to suppress the tumor regrowth during the periods of the study. This suggests that the drug delivery system was successful not just immediately but also for an extended period in stopping the spread and regrowth of residual cancer cells and tumors. The result of this study corroborates that of other studies in which devices were implanted to prevent the re-occurrence of excised TNBC tumors^38^. A scaffold made of 3D-printed poly (lactic-co-glycolic acid), gelatin, and chitosan, containing anti-cancer drugs, reacted to the tumor's slightly acidic conditions when implanted in mice and triggered a controlled, gradual release of the drugs, significantly inhibiting the recurrence and growth of the tumor^79^. This approach is shown to minimize drug side effects and show excellent compatibility with healthy tissues, ensuring no harm while combating the tumor. In a different research study, CS-FA-MBZ nanoparticles loaded with MBZ and targeted with folic acid were characterized and used to create subcutaneous implants in cylindrical form for treating 4T1 triple-negative breast tumors in BALB/c mice^80^.

Compared to conventional drug administration methods, these implants carrying chemotherapy drugs showed notably greater effectiveness. The CS-FA-MBZ implants effectively inhibited the growth and spread of 4T1 breast tumors^80^. Another study has identified an implantable drug loading system for sunitinib nanoparticles at matrix metalloproteinases-response hydrogel (NSMRH), which uses enzyme-sensitive hydrogel as a carrier to load sunitinib nanoparticles^81^. Animal experiments showed that NSMRH combined with radiotherapy could more effectively control the recurrence of subcutaneous xenograft tumors, prolong the survival time, and have no obvious toxicity in nude mice. Animal trials demonstrated that combining NSMRH with radiotherapy was more efficient in managing the recurrence of subcutaneous xenograft tumors, extending survival duration, and exhibiting no noticeable toxicity in nude mice^81^.

**Figure 8:**
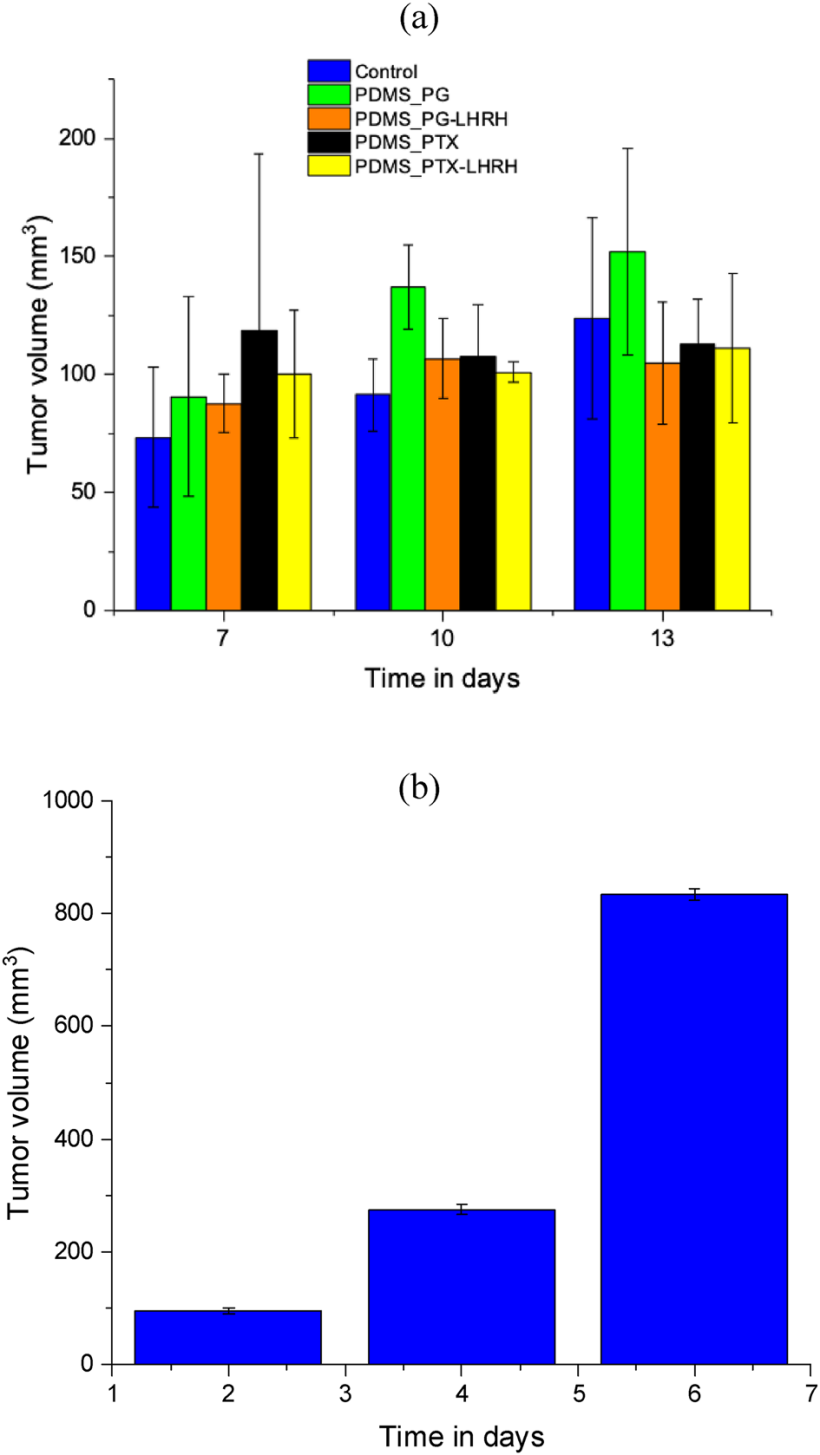
Tumor volumes before and after drug delivery, respectively (**a**) tumor volume before surgical removal and prior treatment, and (**b**) tumor recurrence in control mice (without drug treatment) following surgical tumor removal.

Additionally, tumor recurrence was observed in the control mice that did not receive any drug post-surgical tumor removal. The tumor volumes for the control group were 94.77 ± 5.5 mm^3^ and 276.822 ± 8.8 mm^3^ on the second and fourth weeks, respectively (Fig. 8). By the sixth week, the tumor volume had reached 834.9 ± 10.2 mm^3^, and it's noteworthy that no fatalities occurred during this period. The data suggests a time-dependent increase in tumor volume, highlighting the aggressive nature of triple-negative breast cancer. Earlier reports have shown the therapeutic efficacy of PDMS nanoparticles as drug delivery carriers^82^.

### Trichrome staining

Interest in implanted medical devices continues to rise because of their potential roles in precision medicine and recent advancements in the development and fabrication of new biomaterials^83^. PDMS is very useful in medical implant applications, particularly due to its biocompatibility and hydrophobic nature^84^. In this work, implantable PDMS was employed as a drug delivery system to prevent tumor recurrence following surgery, and Masson’s Trichrome stain was used to monitor tissue regeneration at 2-, 4-, and 6-weeks post-implantation. In a standard Masson's Trichrome procedure, the cytoplasm is dyed pink, muscle tissue is stained red, the nuclei are stained dark brown, and collagen is stained blue^85^.

**Figure 9:**
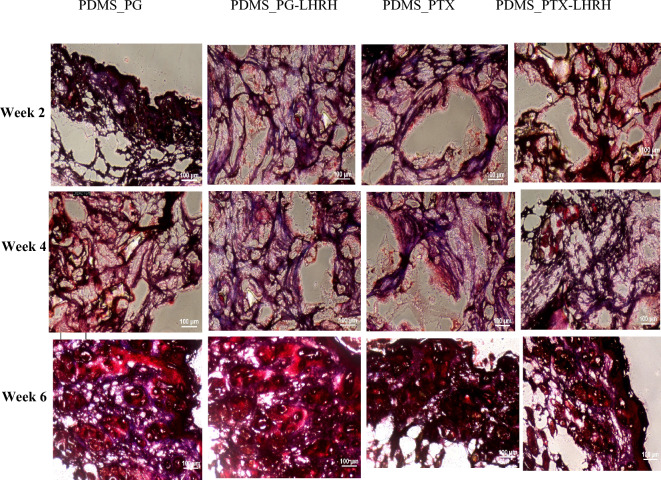
Masson’s Trichrome staining of 4 µm thick sections from 12-week-old female mice treated with different drugs and stained with Masson’s Trichrome (× 100 magnification).

Figure 9 shows that, in all the groups of animals, connective tissues were stained blue, confirming the presence of collagen, which is an indicator of tissue regeneration. This is because the region where the tumors were removed was targeted through drug delivery interventions to stimulate the regenerative potential of tissues coupled with migration of the epidermis over the granulation tissue beneath. Hence, the formation of a mass of cells at the proximal end and the subsequent synthesis of new collagen fibers to fill the space allowed for the regeneration of severed connective tissues. Restoring the morphological and functional characteristics of damaged tissue is the primary objective of tissue regeneration^86^. The results further demonstrated that none of the treated groups experienced new tumor growth, which strongly suggests that completely functional tissue constructs can be regenerated using this system. This result also indicates strong inhibitory effects of the drugs on tumor regrowth**.**

Overall, these results indicated that it is feasible to prevent tumor regrowth in TNBC through targeted and localized drug delivery from a microporous device. Thus, the findings of this investigation motivate future clinical trials and the exploration of the current approach to different types of tumors.

### Liver enzyme assessment

Damage to the liver has an impact on overall health and sometimes puts life in peril. Currently, liver injury is assessed clinically using the liver and serum function indices. One of the main reasons why drugs fail in clinical trials is drug-induced liver damage, a rare but devastating side effect of pharmacological therapy. Traditional biomarkers, particularly serum transaminases and bilirubin, are helpful predictors of cholestatic or hepatocellular liver injury, respectively, but only after significant and possibly permanent tissue damage^87^. Therefore, we evaluated the effect of the drugs on the liver using glutamate dehydrogenase (GLDH) assay.

**Figure 10:**
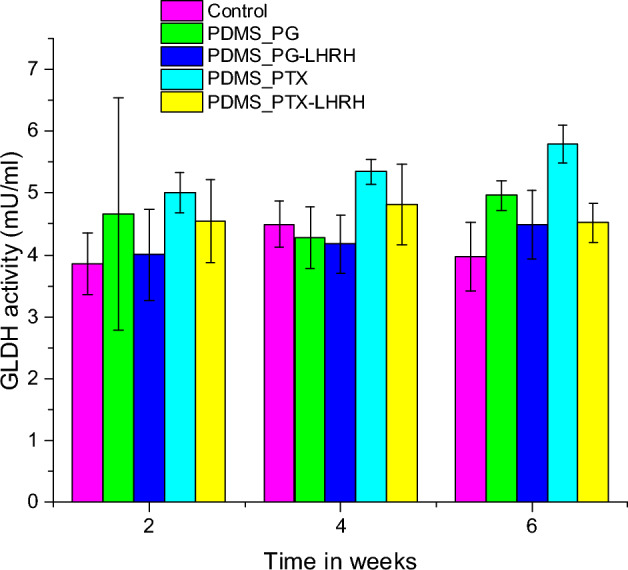
Serum concentrations of glutamate dehydrogenase of nude mice with TNBC tumors treated with PGS-LHRH, PTX-LHRH, PGS, and PTX through a microporous device.

When the integrity of the hepatic membrane is lost, the GLDH enzyme, like ALT, leaks from the injured hepatocyte into the bloodstream and is easily identified using an enzyme activity assay^87^. It has been proposed that GLDH may also be helpful in evaluating mito-toxicity as an initial event during drug-induced liver injury because it is found within the mitochondrial matrix^88^. The mean levels of the GLDH enzymes for the treated and control mice are shown in Fig. 10. Serum GLDH concentrations after two weeks of treatment were not significantly different (p > 0.05) in all groups, including the control group, which had not received any treatment or had been exposed to subcutaneous xenograft tumors.

On week four, the group that received PTX treatment had significantly greater (p > 0.05) serum GLDH levels compared to the other groups, except for group 5, which received PTX-LHRH treatment. Also, group 5 was significantly higher (p < 0.05) than group 3 treated with PG-LHRH. Meanwhile, there was no significant difference (p > 0.05) between group 5 and the rest of the group. On the sixth week, the enzyme levels were 4.96 ± 0.24 mU/ml for the PG group and 4.49 ± 0.56 mU/ml for the PG-LHRH group. The levels for the PTX and PTX-LHRH groups were 5.79 ± 0.31 and 4.53 ± 0.32 mU/ml, respectively. Meanwhile, that of the control group was 3.97 ± 0.56 mU/mL. On the sixth week still, a highly significant decrease in the GLDH enzyme level was noted in mice treated with PTX-LHRH compared with PTX (p < 0.05).

There was no significant difference in enzyme levels between the control and PG-LHRH groups (P > 0.05), but the PG group had a significant increase in GLDH enzyme level compared to the PG-LHRH group (p < 0.05). Furthermore, throughout the treatment, the group that was implanted with the device that had the conjugated drugs did not show significant elevations. Also, the mice treated with paclitaxel had higher GLDH levels throughout the assessment. The detection range of serum glutamate dehydrogenase (GLDH) in the mice is 0.156–10 mU/mL^89,90^.

Serum GLDH activity has been suggested as a liver-specific biomarker of liver damage in humans^91,92^. When the integrity of the hepatic membrane is lost, the GLDH enzyme leaks from the injured hepatocyte into the blood and is easily identified using an enzyme activity assay. The fact that serum GLDH remained within the range of normal enzymatic activity during the whole observation period in all groups suggests that there was no liver damage. This result is consistent with our findings in the liver histological studies, as shown in Fig. 10. Several characteristics of GLDH, a crucial enzyme in the oxidation of amino acids and the formation of urea, make it appealing as a possible biomarker of drug-induced hepatocellular toxicity^93^. Across many species, it is well conserved in terms of its structure, tissue-distribution and function^94^. GLDH has demonstrated potential as a sensitive and reliable biomarker of liver damage in humans^95^. In preclinical trials, GLDH has demonstrated comparable or superior sensitivity and specificity to ALT^96,97^.

Furthermore, throughout the duration of the study, the GLDH levels of the conjugated drugs were observed to be lower than the unconjugated forms, and the enzyme levels of PG-conjugated drugs were lower than the PTX conjugated drugs. These low enzyme levels of the targeted drugs are in line with reports that targeted medication delivery is a promising method of reducing the toxicity of chemotherapy drugs^98^. A significant advantage of targeted chemotherapy is an improvement in bioavailability while minimizing toxicity, which is due to a reduction in off-target accumulation of the drugs.

### Creatinine assay

**Figure 11:**
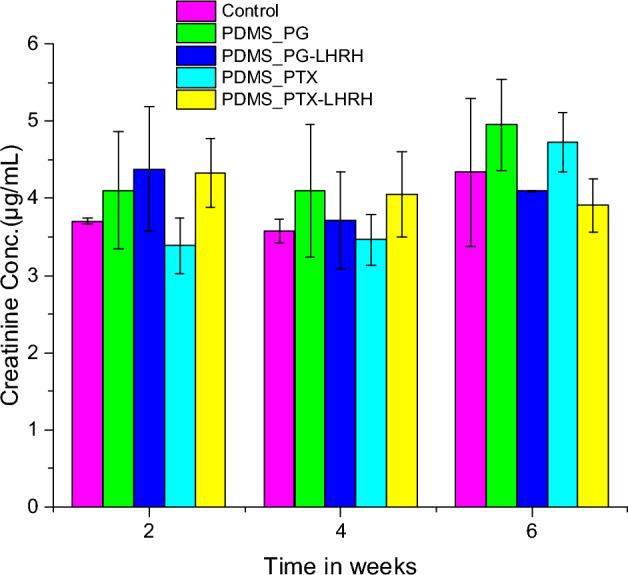
Serum creatinine serum levels of nude mice with TNBC tumors treated with PGS-LHRH, PTX-LHRH, PGS, and PTX through a microporous device.

The results from the creatinine assay are summarized in Fig. 11, and the creatinine levels of the various groups were compared with the control using the least significant difference post hoc test of an ANOVA comparison of means. The comparison of the groups after the 2-week experimental period shows that there was no significant difference between the control group and all the other groups (P > 0.05). Except for the control mice, which had no tumor and were not treated, the other groups (PG-LHRH, PTX, and PTX-LHRH) had significantly higher (P < 0.05) creatinine concentrations compared to group 4 treated with PTX. The lowest creatinine levels were observed in mice that were treated with PG; meanwhile, there was no significant difference (P > 0.05) between groups 2 and 3 treated with PG and PG-LHRH, respectively.

Additionally, when comparisons were made on the fourth week, it was found there was no significant difference (P > 0.05) in the serum creatinine concentration of the control group compared to the other groups. Although not statistically significant, mice treated with PG had the highest levels of creatinine. According to these findings, neither the conjugated nor the unconjugated medicines that were discharged from the microporous device had any negative impact on the kidney. The absence of changes in creatinine levels after four weeks of treatment indicates that kidney function was normal. The reason for the stable creatinine level in all the groups may be due to the specific delivery of the drugs to the tumor site.

A chemical waste product called creatinine is transported through the bloodstream and removed by the kidneys. Creatinine blood levels increase if the kidney's filtration is inadequate. Following a six-week assessment, the creatinine level of the control group was 4.33 ± 0.97 µg/mL, whereas the PG and PG-LHRH groups were 4.95 ± 0.59 µg/mL and 4.10 ± 0.01 µg/mL, respectively. The levels of serum creatinine for the PTX and PTX-LHRH groups were 4.735 ± 0.38 µg/mL and 3.91 ± 0.34 µg/mL, respectively, as shown in Fig. 11. The reported average standard creatinine levels in mice are found to be in the range of 2 to 9 µg/mL^99^.

The serum creatinine concentrations of all the groups, according to our observed result, were all within the standard range. None of the drugs caused any toxicity, and we did not detect significant changes in creatinine levels when the periods of treatment were compared. Also, there was no significant difference in serum creatinine levels (P > 0.05) between the various groups at the end of the 6th week. Comparing mice treated with PTX to animals treated with the PTX-LHRH group, the mice in the latter group showed statistically significant levels of creatinine.

In a similar vein, mice that received PTX-LHRH treatment showed considerably lower levels of creatinine than the PG group. This may be due to the excellent drug delivery performance of the microporous device and the tumor-targeting effects of drugs, which helped in reducing the effect of LHRH on serum creatinine. To the best of our knowledge, there is no scientific report about the effect of PG, PG-LHRH, PTX, and PTX-LHRH on creatinine concentrations. Notably, all these values were within normal limits, indicating that neither hepatotoxicity nor nephrotoxicity was seen in either of the treatment groups. Target drug delivery is a prevalent strategy since targeting the tumor site might produce the intended result with lower doses of each, minimizing unwanted effects. To our knowledge, this is the first study that explores the effect of PTX-LHRH and PG-LHRH on serum creatinine levels.

### Serum SP-D concentrations of female athymic nude mice

SP-D influences cellular processes and pulmonary immunity through direct and indirect interactions with a variety of immune cell receptors^100^. The presence of high circulating SP-D levels has been linked to several lung conditions^101^. Compared to non-TNBC, where the incidence of lung metastasis is just 20%, TNBC can have a 40% lung metastasis incidence rate^102^. When measured in serum, pulmonary surfactant protein D (SP-D) is thought to be a potential biomarker for the functional integrity of the lung and the course of disease^103^. Successive monitoring of SP-D serum levels enables the prediction of the impact of certain treatments on the lungs. The results obtained two weeks after the drug delivery device was implanted revealed that the groups of mice treated with the conjugated drugs had significantly lower (p < 0.05) SP-D concentrations than the groups treated with the unconjugated drugs. Moreover, the SP-D concentrations in the groups that had the conjugated drugs, compared to the control group, showed no significant difference (p > 0.05). Compared to the groups that received the unconjugated drugs, the control groups' SP-D concentrations were significantly lower (p < 0.05).

**Figure 12:**
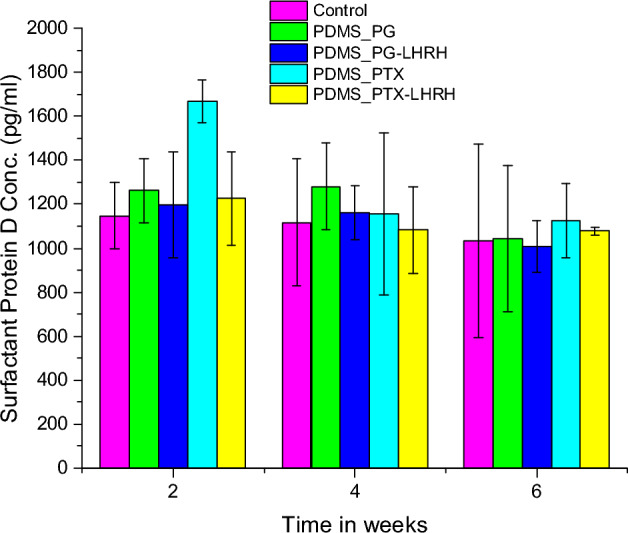
The serum concentrations of surfactant protein D of female athymic nude mice induced subcutaneous xenograft tumors and were treated with PG-LHRH, PTX-LHRH, PG, and PTX delivered from a microporous implantable device.

The findings of the study revealed that, when the SP-D concentrations of each group were compared at the second- and fourth-weeks post-implantation of the drug delivery device, there was no significant difference (P < 0.005). Moreover, there was a progressive decline in serum SP-D levels, particularly in the treated group, from the second to the sixth week. This may be connected to the amelioration of inflammatory-induced structural damage to the alveoli that emerged before the removal of the tumor and the administration of the treatment**.** The serum SP-D levels on the 6th week were 1042.41 ± 333.69 pg/ml in the PG group and 1006.74 ± 117.46 pg/ml in the PG-LHRH group, according to the data obtained 6 weeks after the drug delivery device was implanted (Fig. 12). In the PTX group, the SP-D concentration was 1124.63 ± 170.12 pg/ml, while in the PTX-LHRH group, it was 1078.13 ± 19.43 pg/ml. The control group, which was neither induced with triple-negative breast xenografts nor given treatment, had a serum SP-D concentration of 1033.47 ± 440.25.12. The reference value of Serum SP-D concentration has been reported to be 5000 ± 200 pg/ml in normal C57BL/6 mice^104^, confirming the safe effect of the released drugs on the lungs.

While the serum SP-D levels found in this study were within the acceptable range for this protein, it is important to highlight that the leakage of pulmonary proteins into the circulation is thought to be the cause of high systemic SP-D levels^105^. This permeable anomaly of the alveolar-capillary membrane may be caused by a loss of its structural and functional integrity^106^. In a study performed to check if serum surfactant protein D is elevated and correlated with disease severity^105^, it was stated that IL-6 triggers the release of SP-D into the systemic circulation during a condition of systemic hyper-inflammation and cytokine inflammation. Meanwhile, the serum SP-D levels were significantly lower in the recovery phase for all patients as compared to the acute phase, and during the treatment period. The results of this study demonstrate a similar pattern.

### Histology of the liver

**Figure 13:**
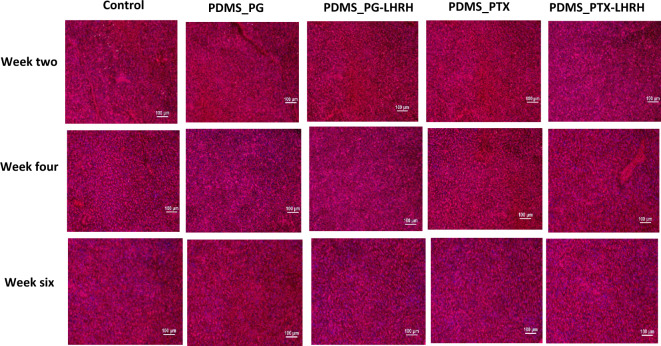
Photomicrographs of mouse liver sections stained with hematoxylin and eosin from each group 6 weeks after drug administration (original magnification 100).

It was necessary to undertake a histological investigation of the liver six weeks after drug delivery to determine the impact of the drugs on the liver due to the significant roles played by the liver in immunity, drug metabolism, and a range of toxic material neutralization and elimination. According to the photomicrograph of the mice's liver examined (Fig. 13), there were no significant, irreversible histopathological alterations or injuries, such as inflammation or necrosis, in either the control group or the treated group. Instead, the portal areas contained the hepatic triad-one or more small branches of the portal vein, a branch of the hepatic artery, and a small bile duct-as well as lymphatic vessels and a very small amount of connective tissue. The lack of morphological alterations suggested that the medications administered did not have any toxicity or detrimental consequences on the liver.

The quantitative toxicity study (liver enzyme assay) results, which revealed that the enzyme levels were all within the reference range six weeks after drug delivery, are supported by this result. The lack of liver-damaging effects observed in this study is because of the drug delivery through the implants. Implants can offer a consistent release pattern with less fluctuation and lower peak values, which may lessen adverse effects^107^. Higher drug concentrations in the intended locations can be achieved by site-specific implantation, which can avoid the oral and peripheral regimens' absorption and distribution phases^108^. Moreover, implantable drug delivery decreases total drug exposure and avoids first-pass metabolism, which is frequently connected to the liver^109,110^.

### Histology of the kidney

**Figure 14:**
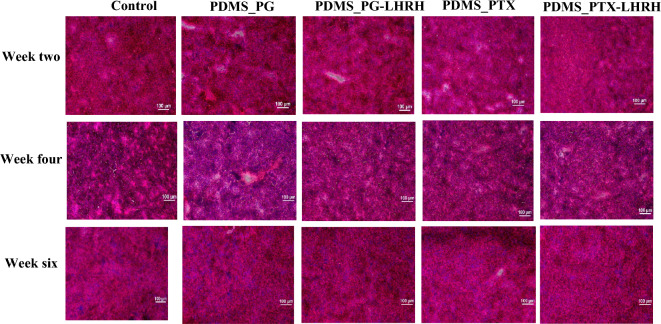
Photomicrographs of mouse liver sections stained with hematoxylin and eosin from each group 6 weeks post-drug delivery (original magnification 100).

The kidney is a histologically complex organ that serves a variety of purposes, such as filtering and regulating minerals, removing metabolic waste products, and maintaining overall fluid balance. Nephrons, which are the glomeruli, tubules, and collecting ducts that make up each kidney, have unique morphological and functional characteristics. The liver histology findings in the treated and control mice were comparable (Fig. 14). This suggests that the drugs did not have any damaging consequences on mice in general. There was no detectable cell infiltration into the dilated distal tubules in any of the groups, but regular and compacted glomeruli as well as many tubules were seen (Fig. 14). The observed safety of the drugs in this study can be attributed to controlled and sustained drug delivery, which extended the duration of drug exposure in the bloodstream. Instead of the rapid and instantaneous elimination seen in conventional drug delivery, this may lead to a more steady and progressive drug clearance by the kidneys. By lowering the peak drug concentrations in the blood, controlled release lessens the chance of surpassing the kidneys' ability to eliminate the drug. This system can maintain efficient therapeutic doses while lowering the risk of renal damage linked to high drug concentrations.

### Histology of the lungs

The lungs are crucial parts of the pulmonary circulation, where the right ventricle of the heart pumps deoxygenated blood through the pulmonary arteries to the lung's alveolar-capillary beds for gaseous exchange. As a result, anything that interferes with the lungs will disrupt its ability to absorb oxygen and exhale carbon dioxide. Thus, this study was carried out to evaluate the impact of treatment on the lungs' histological structures.

**Figure 15:**
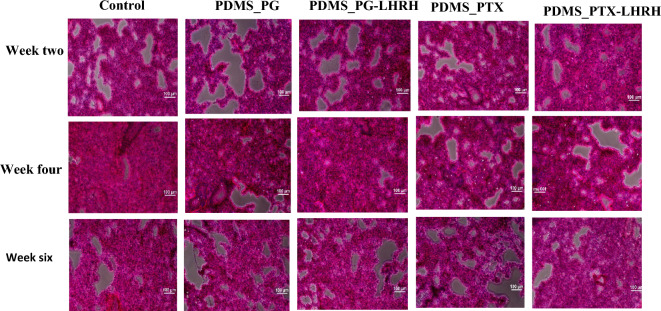
Photomicrographs of mouse lung sections stained with hematoxylin and eosin from each group 6 weeks post-drug delivery (original magnification 100).

The findings of this study (Fig. 15) reveal that there were no differences in the mouse lung's histological structure among the various groups. Hematoxylin and eosin staining showed that neither vascular congestion nor inflammatory cell infiltration were present in the lungs of the individual mouse groups. Moreover, there was no exudation in the lung interstitium or alveolar spaces, and the alveolar wall was unaffected in any way.

Overall, there were no histological changes in the lungs owing to controlled and sustained release through the implants. Controlled release systems could shield the lung epithelium from direct exposure to high levels of drugs, protecting the respiratory epithelium and lowering the risk of damage. Moreover, the mucociliary clearance mechanism plays a critical role in clearing mucus and foreign particles from the respiratory tract; hence, controlled drug release systems can minimize interference with this mechanism and preserve the normal physiological function of the lungs.

## Conclusion

A specialized implantable device using microporous PDMS has been developed to precisely deliver therapeutic agents (LHRH-PTX and LHRH-PG) for the targeted and localized treatment of triple-negative cancer. This PDMS-based system was uniquely designed to be surgically placed in specific anatomical locations within the body after surgical removal of the tumor. This innovative use of PDMS highlights its adaptability and effectiveness in the medical field, enabling the targeted and controlled release of drugs to specific areas. Salient conclusions arising from this study are as follows:

The device demonstrated controlled drug release throughout the period of the study. The drug release kinetics were primarily governed by anomalous transport at the three different temperatures taken into consideration in this work. The therapeutic effects of both conjugated and nonconjugated drugs in vitro demonstrated great promise for drug delivery applications. The in vitro efficacy study showed that the released drugs had remarkable biological activity. The observed therapeutic effects of the conjugated drugs suggest that the conjugation of the drugs with luteinizing hormone-releasing hormone enhanced the activity of the drugs due to specific targeting.

Overall, the in vivo efficacy results indicate that there was no tumor recurrence owing to the microporous structure of PDMS devices, which facilitated localized drug delivery, ensuring that drugs were primarily released at the tumor site. Additionally, the absence of tumor regrowth may be due to effective drug concentrations maintained over an extended period. Even though the strategy employed in this study was effective, it's important to emphasize that combining chemotherapy and hyperthermia within the temperature range of 41 °C to 44 °C is a potential approach to cancer treatment. This strategy has the potential to improve the effectiveness of chemotherapy by sensitizing cancer cells and improving drug delivery to the tumor site. In our current study, we focused solely on understanding the in vitro release profile of the drugs at body temperature (37 °C) and within the hyperthermic range (41–44 °C). However, in our future studies, we aim to explore the combination of our current approach with hyperthermia for TNBC treatment.

The results of the quantitative and qualitative toxicity studies indicate that the organs did not show any hepatotoxicity or pulmonary toxicity, and there were no significant changes in kidney markers. The assessed hepatic, kidney, and lung markers such as serum glutamate dehydrogenase, creatinine, and surfactant D protein levels, respectively, did not show significant elevations, indicating the absence of liver damage. This suggests that the microporous device and the conjugated drugs used in the delivery system have a favorable safety profile for liver, kidney, and pulmonary functions. The absence of organ toxicity may be a key component in drug discovery; hence, this is essential for the clinical translation of the implantable drug delivery system. To support these findings, more investigations and thorough preclinical and clinical investigations are required. For example, in the case of in vitro studies, future work is needed to explore the cytotoxicity and implications of the drug delivery system in more than one breast cancer cell and normal breast cell lines. Moreover, there is a need to carry out hematotoxicity studies in future work to ensure the safety of these treatments, particularly paclitaxel. Hematotoxicity studies will provide a comprehensive understanding of the potential adverse effects of the drugs on the blood and its components. In addition, incorporating pharmacokinetic studies into future work with a larger sample size is essential to obtaining a thorough understanding of how the drugs are distributed in the cells and the body.

In cancer treatment, tumor recurrence poses a serious problem since, even after primary therapy, some cancer cells can survive and eventually cause tumors to grow again. Targeted delivery of drugs with PDMS microporous devices has the potential to prevent tumor recurrence by delivering drugs right to the tumor site, destroying any cancer cells/tumors that remain, and preventing them from proliferating/regrowing. Understanding the interplay between the porous PDMS structure, the drug loaded within it, and the surrounding biological environment is crucial in designing effective and biodegradable drug delivery systems. The four primary ways that polymers used in biomedical devices degrade are enzymatic degradation; hydrolysis (interaction with water in tissues); oxidation (due to oxidants produced by tissues); and physical degradation (e.g., water swelling and mechanical loading and wearing). The degradation can be influenced by the morphology of the pores. Modifying the surface characteristics can affect how PDMS interacts with its surroundings. Smaller pores may impede degradation due to limited access, but larger pores may speed up degradation because they offer more surface area for contact with degrading agents.

## Materials and methods

### List of materials

Details of prodigiosin synthesis^111^. Fetal bovine serum (FBS), penicillin–streptomycin, and Leibovitz's media were bought from the American Type Culture Collection (ATCC, Manassas, VA, USA). Thermo Fisher Scientific (Waltham, MA, USA) provided the paclitaxel, N-hydroxysuccinimide (NHS), 1-ethyl-3-(3-dimethylaminopropyl) carbodiimide hydrochloride (EDC HCl), Alamar Blue Assay kits, Dubecco Phosphate Buffer (DPBS), 12-well plate, and opaque 96-well plates. We purchased dimethyl sulfoxide (DMSO) from Sigma-Aldrich Co. LLC in St. Louis, Missouri, USA. Additionally, Amicon Pro Purification System and 3 kDa Amicon Ultra-4 Centrifugal Filter Units were acquired from Millipore Sigma (Burlington, MA, USA). From Vector laboratories in California, USA, we obtained methanol, alcohol, hematoxylin, and eosin (H and E). A cross-linker and Sylgard® 184 silicone elastomer kit were purchased from Dow Corning Corporation in Midland, USA. Four-week-old athymic Nude-Foxn1nu strain mice from Envigo (South Easton, Massachusetts, USA) weighed an average of 24 g. American Culture Type provided the MDA-MB-231 cell line that was used.

## Methods

### Preparation and characterization of drug-LHRH conjugates

Prodigiosin and paclitaxel were conjugated separately to Luteinizing Hormone-Releasing Hormone (LHRH) for the specific targeting and treatment of triple negative breast cancer (TNBC), as described in previous work^111^.

### Fabrication of drug-loaded implantable device

Polymerization of poly-n-isopropyl-acrylamide (PNIPA)-based hydrogels for biomedical applications was adopted from early works^112–114^. The cytotoxicity of PNIPA-based hydrogels from the early studies warrants the need to encapsulate them into biocompatible compartments like PDMS for localized drug elucidation.

Poly-dimethyl-siloxane (PDMS) was produced by combining a silicone elastomer monomer and a silicone elastomer curing agent at a weight ratio of 10:1. The polymer blend was mixed vigorously for homogeneity. The mixing process generated gas bubbles, which were subsequently degassed with an isotemp vacuum oven (model, 280A, Thermo Fisher Scientific), connected to a high vacuum pump (model, E2M2665503, Edward vacuum) at -25 mmHg for an hour without heating. Sample sugar cubes were properly arranged and separated using aluminum foil inside Petri dishes. The PDMS solution was gently cast on the sugar cubes and cured at 60 °C for 3 h in the oven. The cubes were carefully removed after curing. They were then leached by soaking in distilled water for several days and stored in a desiccator for characterization and further fabrication of the drug delivery device. Specialized blades were utilized to make incisions on the side of the P-PDMS. P(NIPA)–based hydrogels were carefully inserted into the P-PDMS structures' inner core. The complete devices consisted of P-PDMS scaffolds with dimensions of 1.15 mm in width, 1.31 mm in length, and 0.106 mm in thickness. PDMS prepared at a 10:1 ratio was degassed and used to seal the P-PDMS- devices encapsulated with P(NIPA). Curing was achieved at 60 °C for 3 h to ensure complete polymerization. In this way, the porous PDMS device for efficient drug release is formed.

**Figure 16:**
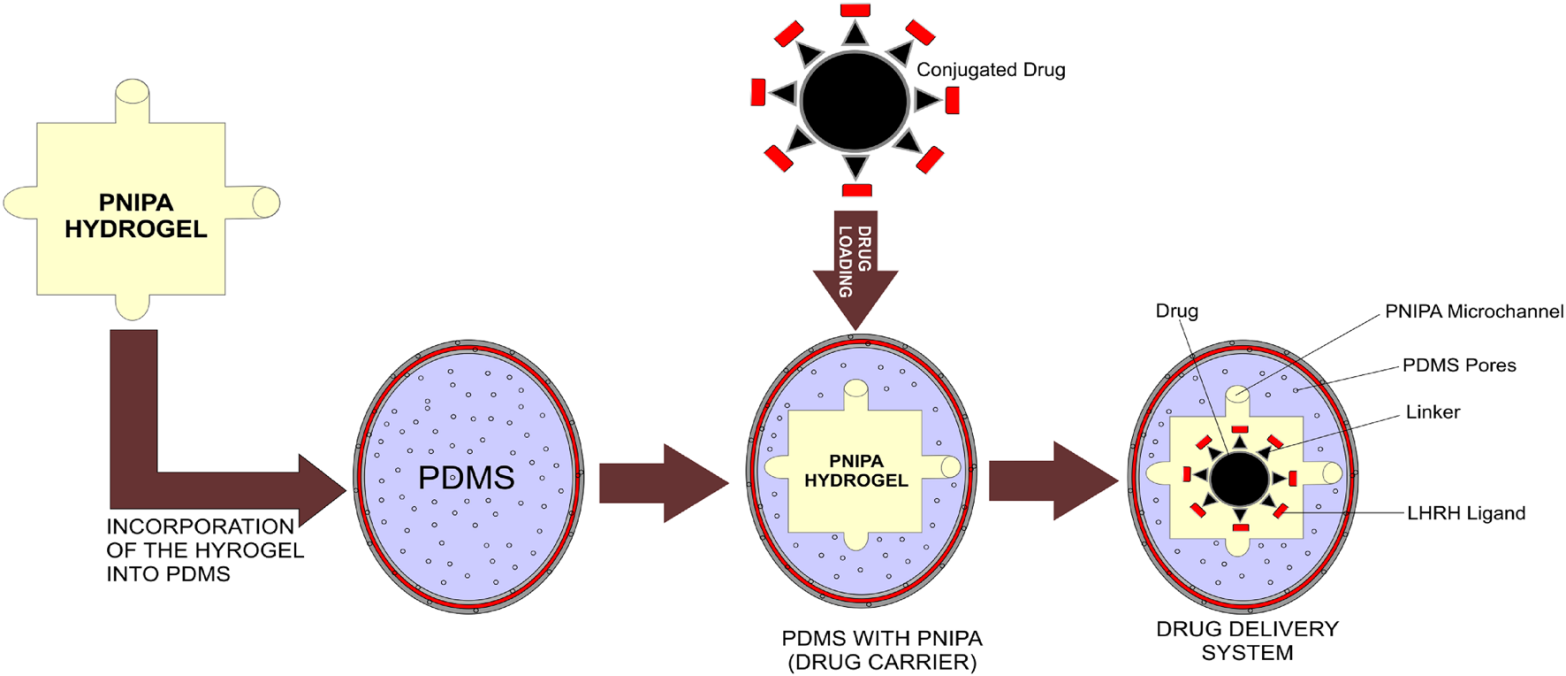
Schematic representation of drug loaded implantable device.

Conjugated cancer drugs (prodigiosin-LHRH, paclitaxel-LHRH) and unconjugated drugs (prodigiosin and paclitaxel) were prepared at working concentrations of 0.5 mg/ml and loaded into the device (as illustrated in Fig. 16) by incubating the prepared drug solutions for 72 h in a refrigerator (in the dark to prevent degradation due to UV light).

### Characterization of PDMS

#### Fourier-transform infra-red spectroscopy (FTIR)

FT-IR spectrometer (Shimadzu, Kyoto, Japan) attenuated with a horizontal attenuated total reflection (ATR) accessory was used to collect Fourier transform infrared (FT-IR) spectra of the porous and non-porous PDMS polymers at wavelengths between 400 and 4000 cm^−1^. Sample peaks were filtered and analyzed for peak matching with the associated spectra.

#### Scanning electron microscopy (SEM)

SEM was used to study the microstructure of the PDMS structures that were used in this study. The specimens were first spin-coated with a sputter coater (EMS Q150R rotary pumped coater). The microstructural details of the PDMS-nanoparticle-based structures were studied using a scanning electron microscope (SEM) equipped with an Oxford Energy Dispersive Spectroscopy (EDS) detector for elemental analysis (JEOL, JSM-7000F Field Emission Scanning Electron Microscope, Tokyo, Japan)^115^.

#### Thermal analysis

The degradation temperature of the fabricated PDMS device was measured using thermogravimetric analysis (TGA) (NETZSCH-Geratebau GmbH, Germany). Each sample, weighing 10 mg, was heated in an Al_2_O_3_ 85µL crucible at a rate of 20 mL/min from 25 °C to 800 °C with 20 mL/min purified nitrogen purging. The Proteus analysis program was used to record and evaluate the data (NETZSCH-Geratebau GmbH, Germany). Differential Scanning Calorimetry (DSC) (NETZSCH-Geratebau GmbH, Germany) was used to measure the amount of heat emitted or absorbed by the materials. Approximately 10 mg of samples were placed in aluminum pans using tweezers. The lids were then sealed. The DSC was characterized in the temperature range between 25 and 400 °C. This was done under a constant flow of nitrogen (20 mL/min). An identical, empty, hollow pan was taken as the reference. The exothermic peaks were analyzed using the thermal analysis software (Proteus) provided with the instrument.

### In vitro drug release studies

After encapsulating 0.5 mg/ml of prodigiosin, paclitaxel, LHRH-conjugated prodigiosin, and LHRH-conjugated paclitaxel in the microporous structures, a 30-day in vitro drug release study was carried out. The in vitro drug release studies were performed at three different temperatures: 37, 41, and 44 °C. The 37 °C corresponds to human body temperature, while 41 °C and 44 °C are regarded as hyperthermic temperatures. The implantable drug delivery systems containing the drugs were immersed separately in centrifuge tubes containing 1 mL of phosphate buffer pH = 7.4, maintained at 37, 41, and 44 °C. Centrifuge tube racks (holding the centrifuge tubes) were then placed on an orbital shaker (Innova 44 Incubator, Console Incubator Shaker, New Brunswick, NJ, USA) that was rotated at 60 rpm. After 24 h intervals, 750 μL aliquots were withdrawn from the tubes after centrifugation at 4000 rpm for 5 min. The tubes were then replenished with 750 μL of fresh PBS before being returned to the incubator shaker for subsequent drug release studies every day for thirty days. The UV–vis spectrophotometer (UV-1900, Shimadzu, Tokyo, Japan) was used to measure the absorbance values of the released drugs in the aliquots at a wavelength of 536 and 230 nm for PG and PTX, respectively. The drug release profile over the 30-day period at different temperatures was analyzed to determine the release rate ancumulative drug release.

### In vitro drug release

The in vitro drug release data was fitted with the Korsmeyer-Peppas model that was used to explain the drug release kinetics. The rate at which a polymeric material elutes its content is described by the Korsmeyer-Peppas model. This model, often known as the power law model, provides a correlation between the time it takes for an active agent to release and its rate of release. This is given by^116,117^:

1 $$\frac{{M_{t} }}{{M_{\infty } }} = kt^{n}$$

where M∞, Mt, t, k, and n, respectively, stand for the quantity of drug released at equilibrium, the amount released in time t, the duration of the drug release, the release rate constant, and the release exponent. The above equation can also be expressed in terms of concentration, with C_t_ being the active agent's concentration at time t and C_∞_ denoted as shown below.

2 $$\frac{{C_{t} }}{{C_{\infty } }} = kt^{n}$$

Taking the natural logarithm of the two sides of Eq. (2):

3 $$In\left( {\frac{{C_{t} }}{{C_{\infty } }}} \right) = Ink + nlnt$$

Hence, in this way, the power law exponent, n, was determined from the slope of plots of ln($C_{t}$/$C_{\infty } ) {\text{versus}} {\text{lnt}}.{ }$.

### Cell lines and cell culture

Human triple negative breast cancer cell lines (MDA-MB-231) were used in this study. The cells were cultured in modified Leibovitz’s L-15 media (Manassas, VA, USA), that were supplemented with 10% fetal bovine serum (ATCC, Manassas, VA, USA) and 100 I.U./ml penicillin/100 lg/ml streptomycin. The cells were incubated at 37 °C under normal atmospheric pressure. They were passaged every 2–3 days when they became confluent.

### Cell viability and drug cytotoxicity

Alamar Blue (AB) reagent was used to study the viability of MDA-MB-231 cell lines (under in vitro conditions) in the log phase of growth. The cytotoxicity of the drugs released from the devices was determined. First, MDA-MB-231 cells were harvested with trypsin–EDTA in the presence of Dulbecco Phosphate Buffer (DPBS). Approximately 3 × 10^5^ cells/well were seeded on cover slips (CELLTREAT, Pepperell, MA, USA) in 12-well culture plates with L15^+^ (Leibovitz’s 15 medium with cell medium supplement of FBS and penicillin/streptomycin).

The devices were sterilized with UV radiation under sterile conditions and washed with sterile water and cell culture-grade DPBS. The drug-loaded devices, which contained drugs at a working concentration of 0.5 mg/ml, showed no signs of leakage. After a 24-h attachment period (of the cells), the microporous drug delivery devices were placed over the culture in the 12-well plates and incubated for 0, 6, 24, 48, and 72 h at 37 °C. After that, the Alamar Blue (AB) reagent (Thermo Fisher Scientific, Waltham, MA, USA) was used to characterize cell viability and cytotoxicity throughout incubation durations. The cell culture media were replenished with 1 ml of 10% AB solution at each time point (in the culture medium). A 100 μl of the solution incubated with AB solution (ABS) was placed into wells of a black opaque 96-well plate after each time point in triplicate (n = 3) (Thermo Fisher Scientific, Waltham, MA, USA).

The percentage AB reduction and the percentage (%) cell growth inhibition were determined, respectively as presented in Eqs. (4) and (5):

4 $$\% Reduction = \frac{{FI_{sample } - FI_{10\% AB} }}{{FI_{100\% R} - FI_{10\% AB} }} \times 100$$

5 $$\% Growth \;Inhibition = \left( {1 - \frac{{FI_{treated} }}{{FI_{untreated} }}} \right) \times 100$$

where FI_sample_ denotes the fluorescence intensity of the cells (treated or untreated), FI_10%AB_ denotes the fluorescence intensity of 10% AB reagent (negative control), and FI_10%R_ is the fluorescence intensity of 100% reduced alamar blue (positive control). The fluorescence intensity of treated cells is denoted by FI_treated_, and the fluorescence intensity of untreated cells is denoted by FI_untreated_.

### Apoptosis assays using flow cytometry

The mechanism of cell death in the breast cancer cells (MDA-MB-231) was determined using flow cytometry. Within 6-well plates, 100 µL of MDA-MB-231 at 3 X 10^5^ cells/mL were added and incubated for 24 h at 37 °C. When the cell confluence was about 80%, the cells were treated for 24 h with microporous membrane devices containing 0.5 mg/ml of prodigiosin, paclitaxel, LHRH-conjugated prodigiosin, and LHRH-conjugated paclitaxel. For apoptosis rate detection, the cells were digested using trypsin without EDTA, washed with PBS, and suspended in binding buffer at a concentration of 1 ~ 5 × 10^5^ /mL. Subsequently, 100 µL of cell suspension was added to a 5 mL flow tube. Then, 5 µL Annexin V-EGFP was pipetted into the flow tube and incubated in darkness for 5 min. A 10 µL propidium iodide (PI) staining solution was applied, followed by the addition of 400 µL PBS. Then, using flow cytometry, all sample flow detection was carried out instantly (FACS Calibur, Model, Becton Dickinson, City, State, USA).

### In vivo animal studies

#### Approval of protocol for animal use

Forty-five Athymic Nude-Foxn1nu strain mice (4 weeks old) with an average weight of 24 g were purchased from Envigo (South Easton, MA, USA). The Institutional Animal Care and Use Committee (IACUC) at Worcester Polytechnic Institute approved these animals for use in this study. All the animals were maintained in accordance with the approved WPI IACUC procedures and guidelines. Researchers also certified all the certification courses via the Collaborative Institutional Training Initiative (CITI program). The mice were housed (three mice per cage) with food and water *ad libetum* with 12 h light/dark cycles.

#### In vivo tumor development

5.0 × 10^6^ MDA-MB-231 human triple negative breast cancer cells (suspended in sterile saline) were injected into the interscapular region (for a stronger angiogenic response) of each mouse to create subcutaneous tumor xenografts. Palpation was used to examine the tumor's development. Tumor volume was calculated by measuring the diameter of the long side (a) and the diameter perpendicular using digital calipers. Tumor volume was calculated using the following modified ellipsoidal formula^118^

6 $${\text{Tumor Volume }}\left( {{\text{TV}}} \right) = \frac{{W^{2} {\text{X}} l}}{2}$$

where L is the tumor's longest axis diameter and W is the longest transverse diameter.

#### Experimental design and tumor growth inhibition studies

Due to pioneering investigations as well as animal welfare concerns, the mice were randomly assigned to three treatment groups. Tumors were allowed to grow for fourteen days after tumor induction and the mice were randomly assigned into five groups consisting of three mice per group. Following isoflurane anesthesia, the tumors were surgically removed from the mice and the microporous device was implanted using IACUC guidelines.

The following groups of *in-vivo* experiments were carried out: group one (0.5 mg/g of paclitaxel in microporous device); group 2 (LHRH conjugated paclitaxel in microporous device); group 3 (0.5 mg/g of prodigiosin in microporous device); group 4 (0.5 mg/g of LHRH conjugated prodigiosin in microporous device); group 5 had the tumor but was not given any treatment, and group 6 was designated as a control group that had neither the tumor nor device injected with vehicle and untreated control and uninfected control. The weights of the mice and their tumor sizes were monitored and measured daily using digital calipers. The mice in each treatment group were euthanized at the completion of the treatment protocol. The tumor tissues, as well as tissues from the mouse's major organs, were then removed from all the mice (kidneys, liver, and lungs) through carbon dioxide euthanasia.

### Biochemical studies

The whole blood obtained from the mice at weeks two, four, and six, was centrifuged at 5000 rpm for 20 min at 4 °C. This was used to obtain the serum for biochemical analysis. All biochemical assays were performed using a microplate reader (Vitros-250, Johnson and Johnson, City, State, USA).

#### Liver enzyme assay

Liver function was evaluated by glutamate dehydrogenase assay as described in the glutamate dehydrogenase activity assay kit (Sigma Aldrich, MO, USA). The master reaction mixture consisting of 82 mL glutamate dehydrogenase (GLDH) assay buffer, 8 mL GLDH developer, and 10 mL glutamate was set up. 100 mL of the master reaction mixture was added to each of the wells containing the samples, thoroughly mixed, and incubated at 37 °C for 3 min. Then, the absorbance was measured at 450 nm. The incubation was continued at 37 °C, while the absorbance was measured at 450 nm every 5 min until the most active sample's value exceeded the highest standard's value (10 nmole/well).

#### Determination of renal function

Nephrotoxicity was assessed by creatinine (CR) assay using a Crystal Chem mouse creatinine assay kit. Exactly 270 µL of sarcosine oxidase solution (reagent CC1) and 8 µL of sample were added into each well of a microplate, thoroughly mixed by repeated pipetting, incubated at 37 °C, and allowed to equilibrate to 37 °C for 5 min. The absorbance was then measured at 550 nm using a plate reader. Then, 90 µL of peroxidase solution (reagent CC2) was added and thoroughly mixed by repeated pipetting, while the absorbance was read again (using a plate reader) at 550 nm after 5 min at 37 °C.

#### Assessment of lung function

Lung function was determined by serum levels of surfactant D protein. The measurement of serum surfactant protein D (SP-D) was done using an enzyme-linked immunosorbent assay (ELISA). SP-D was measured using a commercially available ELISA kit (Model, Endogen, City, MA, USA).

One hundred microliters per well of the 20,000 pg/mL, 10,000 pg/mL, 5,000 pg/mL, 2,500 pg/mL, 1,250 pg/mL, 625 pg/mL, and 312.5 pg/mL Mouse SP-D standard solutions were aliquoted into a precoated 96-well plate, and 100 µL of the sample diluent buffer was added to the control well. Then, 100 µL of each properly diluted sample of mouse serum was added to each empty well. The plate was then sealed with a new adhesive cover and incubated at 37 °C for 90 min. The cover was then removed before discarding the plate content and blotting the plate onto paper towels or other absorbent material.

Thereafter, 100 µL of biotinylated anti-Mouse SP-D antibody working solution was added into each well, and the plate was sealed with an adhesive cover and incubated at 37 °C for 60 min. The plate was washed three times with 0.01 M TBS or 0.01 M PBS, and each time, the washing buffer stayed in the wells for a minute. The washing buffer was discarded, and the plate was blotted onto paper towels. A 100 µL of prepared Avidin–Biotin-Peroxidase Complex (ABC) working solution was added into each well, and the plate was sealed with a new adhesive cover provided and incubated at 37 °C for 30 min. Then the plate was washed five times with 0.01 M TBS or 0.01 M PBS (each time letting the washing buffer stay in the wells for 1–2 min). The washing buffers were discarded, and the plate was blotted onto paper towels. A total of 90 μL of prepared TMB color-developing agent was added into each well, the plate was sealed with a new adhesive cover, and it was incubated at 37 °C in the dark for 15–20 min. Each well received 100 L of prepared TMB Stop Solution. The O.D. absorbance was read at 450 nm in a microplate reader. This was done within 30 min of adding the TMB Stop Solution.

### Trichrome staining

To check for tissue formation within the implanted device, collagen contents were assessed histologically using trichrome staining. Frozen tissue sections were fixed with methanol at 4 °C for 5 min, and then placed in PBS for 10 min. Bouin’s fluid was preheated in a water bath to 56–64 ºC in a fume hood. The slides were then incubated in Bouin’s fluid for 20–40 min, followed by 10 min of cooling. The slides were rinsed in tap water for 5 min and rinsed once in distilled water. Slides were stained with Weigert’s iron hematoxylin for 5 min and rinsed in running tap water for 2 min. Slides were placed in a Briebirch Scarlet/Acid Fuchsin solution for 15 min and subsequently rinsed in distilled water. Slides were then differentiated in Phosphomolybdic/Phosphotungstic acid solutions for 10–15 min, followed by the application of aniline blue solutions for another 5–10 min, and rinsed in distilled water. Acetic acid solution (1%) was applied to the slides for 3–5 min, followed by dehydration in 95% alcohol twice and absolute alcohol twice. Finally, the slides were cleared in Xylene (with 2 changes of Xylene for 5 min) and mounted in synthetic resin.

### Histopathological studies

The histopathological assessment was done in the histology laboratory of the Worcester Polytechnic Institute, Worcester, MA, USA. At the second, fourth, and sixth weeks after implantation of the microporous device containing the drugs, the animals were sacrificed using the WPI Animal Care and Use Committee-approved euthanasia technique. The organs were observed for gross lesions, and then the liver, lungs, and kidneys were collected for histopathological examination. A layer of freezing media was placed onto a specimen chuck, and samples were mounted and allowed to solidify. Cross Sects. (5 m thick) of the fixed samples were cut with a microtome (Leica PM 2125, Germany) and stained with hematoxylin and eosin. Sections were fixed in 80% methanol for 5 min at 4 °C before being washed with phosphate-buffered saline to remove the fixative.

The tissues were then washed in deionized water before being stained with hematoxylin and subsequently incubated for 5 min. After washing the tissues in two changes of distilled water (15 s each), an adequate amount of bluing agent was applied. It was rinsed in two changes of distilled water and dipped in 100% ethanol to remove any remaining stain. The tissues were stained with eosin and incubated for 2–3 min, followed by rinsing in 100% ethanol. The tissues were then dehydrated in three changes of 100 ethanol (1–2 min) and visualized under a light microscope (Magnus, India) to study the microscopic architecture of the organs.

### Data analysis

The Statistical Package for the Social Sciences (SPSS) version 20 and the OriginPro 2017 software package were used to conduct the statistical analysis. The results were presented as mean ± standard deviations. To compare the differences in means among multigroup data, one-way analysis of variance (ANOVA) was used, and the differences between the experimental and control groups were compared with LSD's test. At P ˂ 0.05 was considered significant in all circumstances .

## Reporting of in vivo experiments

The study was reported in accordance with Animal Research**:** Reporting of In Vivo Experiments (ARRIVE) guidelines^119,120^.

### Supplementary Information

Supplementary Information.

## Data Availability

All the data generated and analyzed during this study are included in this article, along with its supplementary information files.
